# Process-related factors associated with disciplinary board decisions

**DOI:** 10.1186/1472-6963-13-9

**Published:** 2013-01-07

**Authors:** Søren Birkeland, Rene dePont Christensen, Niels Damsbo, Jakob Kragstrup

**Affiliations:** 1 Research Unit of General Practice, Institute of Public Health, University of Southern Denmark, J.B. Winsløws Vej 9, Odense C, DK, 5000, Denmark; 2 Department and Research Unit of General Practice, Institute of Public Health, University of Copenhagen, Copenhagen, Denmark

**Keywords:** Case management, General practice, Jurisprudence, Malpractice, Patient complaints

## Abstract

**Background:**

In most health care systems disciplinary boards have been organised in order to process patients’ complaints about health professionals. Although, the safe-guarding of the legal rights of the involved parties is a crucial concern, there is limited knowledge about what role the complaint process plays with regard to board decision outcomes. Using complaint cases towards general practitioners, the aim of this study was to identify what process factors are statistically associated with disciplinary actions as seen from the party of the complainant and the defendant general practitioner, respectively.

**Methods:**

Danish Patient Complaints Board decisions concerning general practitioners completed in 2007 were examined. Information on process factors was extracted from the case files and included *complaint delay,* complainant’s *lawyer involvement,* the number of *general practitioners involved, event duration*, *expert witness involvement, case management duration* and *decision outcome* (discipline or no discipline). Multiple logistic regression analyses were performed on compound case decisions eventually involving more general practitioners (as seen from the complainant’s side) and on separated decisions (as seen from the defendant general practitioner’s side).

**Results:**

From the general practitioner’s side, when the number of *general practitioners involved* in a complaint case increased, odds of being disciplined significantly decreased (OR=0.661 per additional general practitioner involved, p<0.001). Contrarily, from the complainant’s side, no association could be detected between complaining against a plurality of general practitioners and the odds of at least one general practitioner being disciplined. From both sides, longer *case management duration* was associated with higher odds of discipline (OR=1.038 per additional month, p=0.010). No association could be demonstrated with regard to *complaint delay, lawyer involvement, event duration,* or *expert witness involvement*. There was *lawyer involvement* in 5% of cases and *expert witness involvement* in 92% of cases. The mean *complaint delay* was 3 months and 18 days and the mean *case management duration* was 14 months and 7 days.

**Conclusions:**

Certain complaint process factors might be statistically associated with decision outcomes. However, the impact diverges as seen from the different parties. Future studies are merited in order to uncover the judicial mechanisms lying behind.

## Background

In most countries, a disciplinary system handles complaints from patients concerning health professionals, including general practitioners (GPs). The disciplinary systems have the difficult task of weighing a variety of considerations when making decisions in concrete complaint cases. These considerations include strict health professional considerations (concerning the health care actually delivered), but also patient safety interests and professional ethics. The structure of disciplinary systems reflects the judicial safeguarding of these balancing interests. In this regard, the legal rights of the complainants and the involved health professionals persistently play a predominant role. The European Convention on Human Rights states a number of such legal rights (Article 6) including, e.g., tribunal impartiality, the right to be heard and within reasonable time, and the right to legal assistance. Member states of the Council of Europe, like Denmark, are party to the Convention and are obliged to address the protection of fair trials.

In Denmark, a specialised board considers patient complaints about the professional conduct of named health care professionals who are authorised by the Danish National Board of Health. The board is separated from the compensation system and patients only seeking monetary compensation are redirected to the Danish National Health Insurance Association (“Patientforsikringen”). Complaints about the level of service (e.g. unsatisfactory waiting times) are sent to the regional health authority. Furthermore, by the introduction of an additional “Patientombuddet” institution, recent revisions of the Danish system aim at enhancing the possibilities of complaining about courses of health care rather than about named health professionals. As it appears, the complaint system is multifaceted. Therefore, local public advice and complaints advocacy service offices have been established in order to guide people wishing to make a complaint through the different systems and help is given to forward complaints to the right authority.

In January 2011, the board handling complaints about professional conduct was renamed “the Danish Disciplinary Board” (from “The Danish Patient Complaints Board”, DPCB), and some organisational changes were implemented. For the most part the case management procedure is, however, unaltered. The procedure is as follows: After receiving a complaint from the patient concerned or his/her relatives about one or more named health professionals the case is clarified by the secretariat. In this regard, a hearing takes place and the health professional is asked to provide a statement in response to the complaint. Subsequently, the secretariat makes a proposal for a decision. Typically, in those cases not only concerning patients’ formal legal rights, the proposal is based upon evaluations made by the board’s expert witnesses. For instance, complaints against GPs are assessed by GP experts. The board’s decision is made by a five-person committee headed by a chairperson who is a judge. The main disciplinary remedy is a discipline expressed through means of issuing either “criticism” or – until 1 January 2011 – the mildest reaction “dispute of professional conduct”. The latter measures imply that the health professional(s) involved, the complainant, and other relevant partakers receive the board’s disapprobation of the health professional by letter. In case of repetition, a criticism may result in public announcement of the health professional’s name, and – in a few cases – withdrawal of the health professional’s authorisation to practice. Other possible, but rare, sanctions comprise issuing a discipline with injunction, or bringing the health professional concerned for the prosecuting authority. In 2007, the DPCB made 2387 decisions and approximately one fifth concerned treatments in general practice [1].

The disciplinary system has been under an ongoing revision in order to optimise the judicial process. The judicial interests of the parties have been safeguarded by regulations in relation to different process factors (i.e. procedural issues from the medical event concerned until complaint decision). Such process factors considered of importance to the parties include for example expert witness involvement and time limits for complaining. It has been continuously debated whether the complaint system pays enough attention to the involved parties’ legal rights. In this respect, there are two major angles: the side of the complainant party, who complains about one or more health professionals, and that of the individual health professional who receives a complaint.

Presumably complainants seek to improve their complaints’ possibilities of being declared justified (thereby disciplining health care staff) when engaging a lawyer. Likewise, a priori, if the period of time to be assessed is extended, or more health professionals are complained about, the likelihood of identifying some health professional negligence resulting in discipline would possibly increase. This would favour the complainants. Contrarily, it may be hypothesised that due to for example increasing difficulties with satisfying the burden of proof, delayed complaints are less prone to be declared justified. Likewise, it has been claimed that sanctions are less likely if the case is assessed by a peer expert witness [2]. Anyhow, we have limited empirical knowledge as to what extent such process factors are on the whole related to the likelihood of cases resulting in discipline. Reasonable development of the quality and efficiency of patient complaint structures requires such knowledge to inform the legislators and organisers about what really matters during the patient complaint process. Using decisions against Danish GPs, the objective of this study was to investigate what process factors are statistically associated with decisions on discipline as seen from the sides of both the complainant party and the defendant (GP) party.

## Methods

### Study database and population

This study is a part of a larger register-based study concerning disciplinary board decisions towards Danish GPs in 2007. In this cohort study, the board decisions were analysed with regard to a number of process factors in order to compare decisions on discipline (“criticism” or “dispute of professional conduct”) with those not resulting in discipline.

### Data collection

Paper files related to all DPCB decisions in 2007 concerning general practice were reviewed. Decisions were treated both as compound decisions (in some cases involving more GPs) and as separate decisions against individual GPs. In a compound decision some GPs might be disciplined and others might not.

The following information was obtained: *Complaint delay* (time from the medical event concerned until filing the complaint), *lawyer involvement* by the complainant (judicial expertise used to e.g. formulate the complaint), the number of *general practitioners involved*, and the *event duration* concerned (duration of health care episode). With regard to *Complaint delay,* for practical reasons the time interval from the last date of the health care event until registration within the disciplinary board was used. Obviously this time comes after the time of complaining. The date of registration was, however, considered useful because it was unfailingly available in every case and is most probably closely tied with the time of producing the complaint.

The involvement of an *expert witness* and *case management duration* (from the date of registration of the complaint within the disciplinary board until the date of decision) were included as independent variables. Finally, information was gathered on *decision outcome* (dichotomised into “discipline” or “no discipline”).

### Analyses

Factors associated with discipline were identified by means of a multiple logistic regression model. The dependent variable in the model distinguished decisions on discipline from decisions without discipline. Odds ratios (ORs) for cases resulting in discipline with regard to the characteristics (independent variables) mentioned above were estimated. First, the unit used for the statistical analysis was compound decisions relating to the entire complaint filed by the complainant and sometimes including separate decisions about more GPs. In this connection outcome was considered as a “discipline” if *at least one* of the involved GPs had been disciplined; this corresponds to the notion of a complaint being declared ‘justified’. In the second statistical analysis, the unit used was decisions about separate GPs (as seen from the individual GP’s side) again dichotomised into discipline or no discipline. When analysing the separate decisions, clustering at the case level was taken into account by robust estimation. P-values < 0.05 were considered statistically significant. The analyses were undertaken using STATA®, release 11.1.

### Ethical approval

This study was approved by the DPCB and the Danish Data Protection Agency (Licence: 2008-41-2875). In Denmark, registry-based studies do not by law require ethical approval from the local research ethics committee.

## Results

In 2007, 427 compound decisions were made concerning general practice. Sample characteristics are outlined in Table 1.

**Table 1:** Process factors and outcomes in compound complaint decisions (n=427)

**Case process factor**		**[Range]**
Complaint delay, mean	3 months, 18 days	1 day – 47 months, 5 days
Lawyer involvement	20 (5%)	
General practitioners involved, mean	1.33	1-4
Event duration, mean	4 months, 6 days	1 day – 83 months, 5 days
Case management duration, mean	14 months, 7 days	2 months, 3 days – 72 months, 5 days
Expert witness involvement	393 (92%)	
*Decision outcome*		
Discipline *	114 (27%)	

(*) one or more GPs disciplined (criticism or professional conduct disputed, see text).

Most cases (n=338, 79%) only involved one GP. In 55 cases (13%), the number of *general practitioners involved* was two. In 18 cases (4%) 3 GPs were involved, and in the remaining 16 cases (4%) 4 GPs were involved. In 45 cases (11%) one or more non-GPs were involved, most frequently hospital doctors, non hospital specialists (e.g. ear, nose, and throat specialists), and nurses.

The compound decisions concerned 571 separate decisions relating to individual GPs. The association between individual GPs being disciplined and process factors is shown in Table 2.

**Table 2:** Process factors associated with being disciplined as a general practitioner (n=571)

**Discipline**		**OR**	**P**	**95% Confidence intervals**
*Complaint initiation*				
Complaint delay (months)		0.988	0.521	0.954-1.024
Lawyer involvement	No	1		
	Yes	1.257	0.633	0.491-3.216
*Complaint demarcation*				
General practitioners involved		0.661	0.000	0.524-0.835
Event duration (months)		0.996	0.675	0.977-1.015
*Complaint decision*				
Case management duration (months)		1.038	0.010	1.009-1.069
Expert witness involvement	No	1		
	Yes	1.366	0.452	0.606-3.077

When the number of *general practitioners involved* in a complaint case increased, the odds of discipline decreased for the individual GP concerned (OR=0.661 per additional GP involved, p<0.001). Conversely, when analysing the association between process factors and discipline in compound decisions (from the side of the complainant party, table not shown), no statistical association could be detected between the number of *general practitioners involved* and odds of the compound decision resulting in at least one of the litigated GPs being disciplined. In both analyses, however, long *case management duration* was associated with increased odds of discipline. Hence, a six-month prolonged *case management duration* was associated with 26% increased odds of the case resulting in a decision on discipline (p=0.010, 28% in compound decisions, p=0.011). No association could be demonstrated with regard to *event duration, complaint delay, expert witness involvement,* or complainant’s *lawyer involvement*. Even when taking clustering into account in separate decisions, the association between decision outcome and number of *general practitioners involved* and *case management duration*, respectively, was statistically significant.

## Discussion

The key findings of this study are an association between a rising number of *general practitioners involved* and decreased odds of being disciplined as an individual GP and between longer *case management duration* and increased odds of discipline.

The present study covers all Danish complaint cases involving GPs completed during one year and is based on register data and case management files, which are likely to be complete and reliable. It should be kept in mind that, despite the inclusion of nation-wide data, only a limited number of cases involving a lawyer came up and this has a deflating impact on statistical power. The deflation of statistical power may also be of importance regarding the use of expert witnesses, because very few decisions were made without an expert witness.

Furthermore, when comparing with the situation in other countries it should be kept in mind that the material only concerns complaints about the GP’s professional conduct. As mentioned above, complaints only seeking compensation or complaints about the level of service are not dealt with.

With the increased complexity of health care, risks are not only limited to the performance issues but may also relate to issues regarding, e.g., coordination and communication. Therefore*,* a parallel system has been established, where complainants may file a complaint to the “Patientombuddet” with regard to concrete *health care* without intending named health professionals to be disciplined. In those cases, it may be concluded that the health care provided as such was criticisable. The complainant may subsequently file a complaint to the disciplinary board against those named health care providers involved, in order to clarify if any named health professionals should receive a disciplinary sanction as well.

Only limited comparable literature exists concerning the proportion of patient complaints leading to a sanction. However, the present findings that 27% of cases results in a disciplinary action seems reasonably in agreement with previous recordings from the Netherlands that, during a 20-year period, roughly one fifth of complaint cases was declared justified [3].

Likewise, the association between health disciplinary process factors and decision outcomes has attracted little research attention. The only study existing on the relation of process factors to decision outcomes is a Japanese analysis of medical malpractice case decisions [4]. As in the present study, lengthy cases were shown to be associated with decisions in the patients’ favour. The causes of this association might be numerous, but the most likely reason might be that straightforward (short-duration) cases are typically those with limited likelihood of negligence, while the cases resulting in discipline may be more complicated, generally requiring a thorough (long-lasting) case management.

Prolonged case management is demanding for all the involved parties in disciplinary proceedings – not least the defendant. Although (repeated) hearings aim to safeguard legal right requirements (see above), case prolongation may be a hardly avoidable drawback. Still, case management should agree with for example the reasonable time requirement according to the European Convention on Human Rights, Article 6. In this regard, it is noticeable that the increased odds of disciplining the defendant with case management prolongation run parallel to case durations up to 6 years.

It has previously been argued that the involvement of lawyers on behalf of patients may increase the possibility that breaches of standards of practice are clarified, because lawyers to a higher extent bring forth written protocols and standards [5]. However, the present study could not verify any statistical association with decision outcome. One major reason may simply be a lack of statistical power. Nevertheless, lawyers may perhaps only be able to contribute little to the examination of complaint cases. As mentioned above, the disciplinary board has a duty to independently examine the case and maybe therefore a legal representative was involved in no more than one out of twenty complaint cases. Hagihara et al. [4] suggested that only few lawyers have sufficient experience in medical malpractice litigation, and not least in Denmark there is little tradition among lawyers to concentrate their business on health professional disciplinary proceedings.

Even though international guidelines have been issued to ensure expert witness impartiality [6,7], Lens and van der Wal have highlighted the possible mechanism among health professionals of covering up dysfunction in a so-called “conspiracy of silence” [2]. Correspondingly, a Dutch study recently demonstrated that more than one third of “Healthcare Consumer Panel” members had no confidence in the disciplinary proceedings and their independent status [8]. Another Dutch study showed, however, a decreased proportion of complaint cases resulting in discipline after including more lawyers and fewer fellow professionals on the disciplinary board [9]. Fellow expert witnesses are not appointed in order to cover their colleagues and, from a statistical point of view, do not appear to do so. The present study could not document any statistically significant association between fellow professional involvement and the odds of discipline.

Anyhow, the disciplinary board’s preparation of the case is highly dependent on the initial demarcation of the complaint case provided by the complainant. From the complainant’s point of view, no association could be demonstrated between involving a larger group of GPs in the complaint and the odds of disciplining at least one GP. Some would interpret this finding to be satisfactory; the opposite result could suggest an unwarranted element of fault finding. Though, from the point of view of the GP concerned, the demarcation of the number of *general practitioners involved*, contrary to the demarcation with regard to *event duration* and *complaint delay,* was not only trivial. Hence, the study suggests that being involved together with other GPs protects against discipline. In other words, the instinctive sense of relief if realising being litigated in plurality might be substantiated in a statistical counterpart. The mechanisms might be numerous. Complaints against more health professionals might sometimes indicate ‘shooting from the hip’ or hardly transparent system matters. In this regard, the above-mentioned new “Patientombuddet” institution should be mentioned as it allows for less specifically outlined complaints to be handled (e.g. without reference to named health professionals). It is, however, crucial that the analyses do not take into account the content of the complaint material and hence lack the data necessary to further inform about the basis of the statistical findings.

## Conclusions

The analyses of the present article offer insight into some statistical associations between complaint cases resulting in discipline against health professionals and a number of measurable process factors. The impact of the process factors, however, diverged as seen by the complainant party and the defendant party, respectively. Statistically significant associations only existed between being litigated in plurality and decreased odds of discipline, and between prolonged case management and increased odds of discipline.

Optimal complaint case management is an important goal, because complaint cases are very resource-demanding for the involved parties. Statistical analyses on disciplinary process factors might offer valuable information on issues of key judicial impact. For instance, the apparently different odds of being disciplined when litigated alone and jointly, respectively, raise concerns about whether these categories should be offered a differentiated means of legal advice when navigating the complaint process. Likewise, the association between decision outcomes and process prolongation might necessitate the introduction of specified and intensified process measures when reasonable time requirements are endangered.

Given the high financial and human costs associated with the complaint case process, future studies concerning the judicial mechanisms lying behind the suggested associations are needed in order to optimise the handling of complaint cases, simultaneously preserving the involved parties’ legal rights.

## Abbreviations

GP: General practitioner; OR: Odds ratio; DPCB: Danish Patient Complaints Board.

## Competing interests

The authors declare that they have no competing interests.

## Authors’ contributions

The study was conceived by SB, JK, ND, designed by SB, ND, and JK, data analysis was performed by SB and RDC, data interpretation was done primarily by SB, ND, and JK, and drafting of the paper was done jointly by SB, ND, and JK with substantial input from RDC. All authors revised the paper and approved the final version.

## Pre-publication history

The pre-publication history for this paper can be accessed here:

http://www.biomedcentral.com/1472-6963/13/9/prepub
